# Recruitment Strategies for a Randomised Controlled Trial Comparing Fast Versus Slow Weight Loss in Postmenopausal Women with Obesity—The TEMPO Diet Trial

**DOI:** 10.3390/healthcare6030076

**Published:** 2018-07-06

**Authors:** Michelle S.H. Hsu, Claudia Harper, Alice A. Gibson, Arianne N. Sweeting, John McBride, Tania P. Markovic, Ian D. Caterson, Nuala M. Byrne, Amanda Sainsbury, Radhika V. Seimon

**Affiliations:** 1 The Boden Institute of Obesity, Nutrition, Exercise & Eating Disorders, Sydney Medical School, Charles Perkins Centre, The University of Sydney, Camperdown, NSW 2006, Australia; michelle.hsu@sydney.edu.au (M.S.H.H.); claudia.harper@sydney.edu.au (C.H.); alice.gibson@sydney.edu.au (A.A.G.); arianne.sweeting@sydney.edu.au (A.N.S.); johnpresmb@hotmail.com (J.M.); tania.markovic@sydney.edu.au (T.P.M.); ian.caterson@sydney.edu.au (I.D.C.); 2 Metabolism & Obesity Services, Royal Prince Alfred Hospital, Camperdown, NSW 2050, Australia; 3 School of Health Sciences, College of Health and Medicine, University of Tasmania, Launceston, TAS 7250, Australia; nuala.byrne@utas.edu.au

**Keywords:** weight loss, clinical trial, diet—reducing, recruitment, obesity

## Abstract

Current research around effective recruitment strategies for clinical trials of dietary obesity treatments have largely focused on younger adults, and thus may not be applicable to older populations. The TEMPO Diet Trial (**T**ype of **E**nergy **M**anipulation for **P**romoting optimal metabolic health and body composition in **O**besity) is a randomised controlled trial comparing the long-term effects of fast versus slow weight loss on body composition and cardio-metabolic health in postmenopausal women with obesity. This paper addresses the recruitment strategies used to enrol participants into this trial and evaluates their relative effectiveness. 101 post-menopausal women aged 45–65 years, with a body mass index of 30–40 kg/m^2^ were recruited and randomised to either fast or slow weight loss. Multiple strategies were used to recruit participants. The total time cost (labour) and monetary cost per randomised participant from each recruitment strategy was estimated, with lower values indicating greater cost-effectiveness and higher values indicating poorer cost-effectiveness. The most cost-effective recruitment strategy was word of mouth, followed (at equal second place) by free publicity on TV and radio, and printed advertorials, albeit these avenues only yielded 26/101 participants. Intermediate cost-effective recruitment strategies were flyer distribution at community events, hospitals and a local tertiary education campus, internet-based strategies, and clinical trial databases and intranets, which recruited a further 40/101 participants. The least cost-effective recruitment strategy was flyer distribution to local health service centres and residential mailboxes, and referrals from healthcare professionals were not effective. Recruiting for clinical trials involving postmenopausal women could benefit from a combination of recruitment strategies, with an emphasis on word of mouth and free publicity via radio, TV, and print media, as well as strategic placement of flyers, supplemented with internet-based strategies, databases and intranets if a greater yield of participants is needed.

## 1. Introduction

Obesity is a major healthcare burden for both individuals and society, associated with adverse consequences in multiple aspects of wellbeing and quality of life [1,2]. Having a higher body mass index (BMI) is a key risk factor for various non-communicable chronic diseases including cardiovascular disease, type 2 diabetes mellitus and certain cancers [3]. Lifestyle interventions which incorporate dietary and/or physical activity modifications have been demonstrated to be effective in eliciting reductions in body weight and associated risk factors in individuals with overweight and obesity [4]. However, physiological and hormonal changes during the life course, in conjunction with behavioural and lifestyle factors, can promote weight gain and pose challenges to achieving weight reduction goals. A key example are women who have undergone the menopausal transition, which can result in shifts in body composition and fat deposition which are associated with health risk factors in this population group [1]. Hence, there is scope to undertake clinical trials of dietary obesity treatments in postmenopausal population groups.

With any clinical trial, including those of lifestyle-based interventions, recruitment is a crucial determinant of the trial’s success. It has been reported that up to 50% of randomised controlled trials in health and healthcare research fail to recruit their target number of participants, and only half of the trials that successfully reach their target do so within their original timeframe [5]. Current research around effective recruitment strategies in weight loss and nutrition intervention trials have largely been focussed with younger adult groups and thus may not be applicable to older populations [5,6,7,8]. The effectiveness of specific recruitment methods varies depending on characteristics of the population group, such as age, sex and ethnicity, as well as other sociodemographic factors [6,7,8]. Research into recruitment of older people to date has largely focused on clinical trials conducted in primary care, which have traditionally drawn on cancer registries and aged centre facilities to recruit participants. Thus, knowledge about effective ways to recruit free-living non-clinical populations into community-based clinical trials has been limited due to a lack of systematic collection and reporting of recruitment data [9]. Compared to clinical populations, community-based trial participants may be more challenging to recruit due to their diverse behaviours and characteristics. Insights into successful methods for recruitment of such populations would likely benefit research regarding lifestyle-based treatment of obesity and other chronic conditions, as these trials often require non-clinical populations [9,10].

The current paper describes and assesses the relative effectiveness of the recruitment strategies that were used to enrol participants into the TEMPO Diet Trial (**T**ype of **E**nergy **M**anipulation for **P**romoting optimal metabolic health and body composition in **O**besity). In light of the above-mentioned challenges of recruitment for clinical trials, our evaluation of the multi-strategy recruitment process used for the present trial will not only provide insights into the effectiveness of various recruitment strategies, it will also provide information to obesity and health researchers in planning more cost-effective recruitment strategies (both in terms of time and monetary costs), thereby helping to improve the time, resource and financial efficiencies of health research.

## 2. Methods

### 2.1. Ethics

The TEMPO Diet Trial is a randomised controlled trial designed to assess the long-term effects of fast versus slow weight loss on body composition and cardio-metabolic health in postmenopausal women aged 45–65 years with a BMI of 30–40 kg/m^2^. Ethical approval was obtained from the Sydney Local Health District, Royal Prince Alfred Hospital Human Research Ethics Committee. The trial was prospectively registered with the Australian New Zealand Clinical Trials Registry (ANZCTR Reference Number 12612000651886). The inclusion and exclusion criteria for the TEMPO Diet Trial, and the screening methods used to investigate them, are listed in Table 1.

### 2.2. Recruitment of Potential Participants

Multiple strategies were used to recruit participants into the trial over a 3-year period (Table 2). The recruitment strategy mentioned in the prospective participant’s initial email or telephone expression of interest was determined to be the source of trial information which ultimately led to their decision to contact the research team. If prospective participants did not indicate their source of information about the trial in their first communication with our team, this was asked by the research team in subsequent correspondence.

### 2.3. E-mail Screening

Expressions of interest in participation, and subsequent correspondence, were managed through a trial e-mail account. A generic response, which doubled as an ‘e-mail screening’, was e-mailed to all enquirers. It included the following questions, which addressed 6 key eligibility criteria:

1. Do you live in the Sydney metropolitan area?
2. Are you female?
3. Are you 45–65 years of age?
4. Are you at least 5 years postmenopausal?
5. Are you free from diabetes?
6. Do you have a body mass index (BMI) of 30–40 kg/m^2^?

Attached to the e-mail screening message was a participant information sheet with details of the trial purpose and procedures, and the e-mail also contained an invitation to undertake a telephone screening if the recipient was still interested in participating in the trial and could answer ‘Yes’ to all of the above 6 questions.

### 2.4. Telephone Screening

Eligible respondents to the e-mail screening were invited to participate in a 20-min telephone screening, during which they were given details of the trial and were asked a series of standardised questions based on the full eligibility criteria. The standardised questions were asked in the order of the inclusion and exclusion criteria as listed in Table 1. If the potential participant did not meet a particular criterion, the telephone screening was terminated, and that criterion was recorded as the reason for ineligibility. If the individual was classified as eligible after completing the telephone screening, a 1-h face-to-face medical screening was arranged.

### 2.5. Face-To-Face Medical Screening

Written informed consent was obtained, prospective participants were asked to complete and sign a magnetic resonance imaging (MRI) safety questionnaire, and a face-to-face screening interview and medical tests were conducted (or results from any recent medical tests were examined), in order to fully assess the eligibility of prospective participants. They were asked to provide (either in person at the face-to-face medical screening, or via e-mail), a copy of their results from any bone density scans of the hip that they had undergone in the past 2 years, and any fasting blood tests that they had undergone in the past 6 months. If no bone density results were available, a bone density scan of the left hip was conducted in our facility. The bone density scan results were reviewed by one of our endocrinologist co-authors (A.N.S. or T.P.M.) to check for osteoporosis. Any available fasting pathology results were also reviewed by T.P.M. or A.N.S. to determine whether tests for the following conditions had been undertaken, and—if yes—whether the results showed the prospective participant was eligible for inclusion. Specifically, this included full blood count (to exclude extreme anaemia that could be exacerbated by the fast weight loss diet to be used in the trial), plus circulating concentrations of thyroid stimulating hormone (to exclude hyperthyroidism or hypothyroidism), glucose and glycosylated haemoglobin (HbA_1c_, to exclude diabetes mellitus), as well as markers of hepatic and renal function (to exclude hepatic or renal impairment, which may render fast weight loss unsuitable). If any or all of the required fasting pathology results were not available, a pathology request form from a pathology service accredited with the National Association of Testing Authorities (NATA; Douglas Hanly Moir, Sydney, Australia) was given to prospective participants, including request for any fasting pathology tests that were missing from tests undertaken in the past 6 months. Prospective participants were reimbursed for the cost of any pathology tests requested at the face-to-face medical screening, if they were found to be ineligible for the trial, or—if eligible for the trial—at 36 months, once they had completed all trial outcome measurements.

### 2.6. Interventions

Eligible participants were randomised to either a total meal replacement diet (KicStart^TM^ meal replacement shakes and soups from Prima Health Solutions Pty Ltd., Brookvale, NSW, Australia) involving severe energy restriction (target range for energy restriction was 65–75% relative to estimated energy expenditure) for 16 weeks, or until a BMI of no lower than 20 kg/m^2^ was reached, whichever came first, followed by slow weight loss (as for the SLOW intervention) for the remaining time up until 52 weeks (“FAST” intervention), or they were randomised to a prescribed food-based diet involving moderate energy restriction (target range for energy restriction was 25–35% relative to estimated energy expenditure) for a total of 52 weeks (“SLOW” intervention). A protein intake of 1 g per kg of actual body weight per day was prescribed for both interventions. Full details of the dietary interventions have been published previously [14].

The number of participants to be recruited for the trial (*n* = 100) was determined based on power calculations, allowing for up to 20% attrition as seen in previously-published weight loss interventions [15,16,17].

## 3. Results

### 3.1. Participant Recruitment

**Figure 1:**
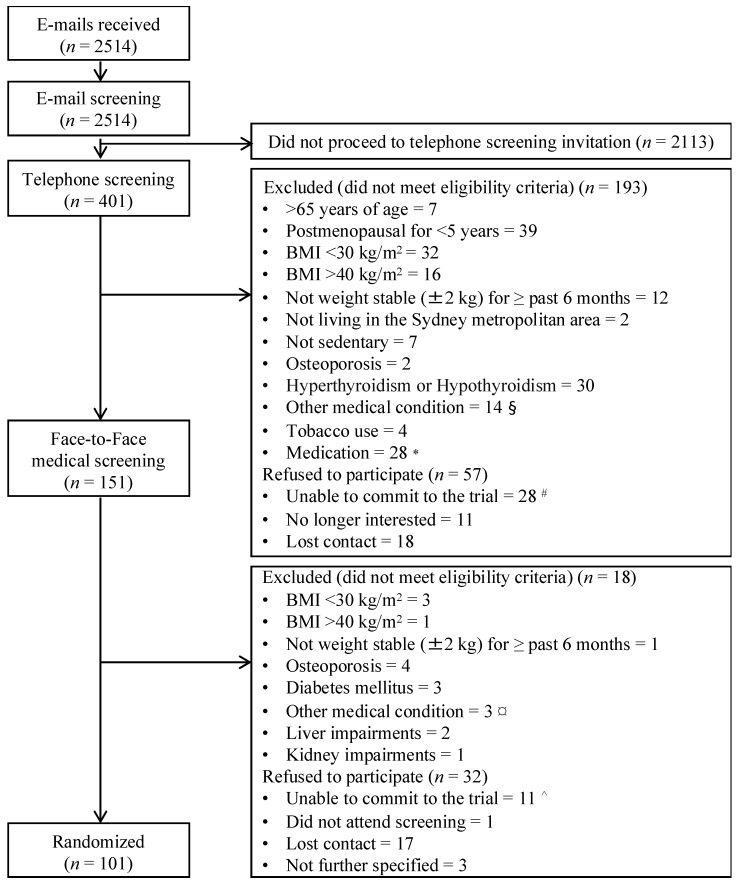
Recruitment flowchart. BMI = body mass index. * Anti-depressants = 21, anti-anxiety = 2, anti-epileptic = 1, anti-diabetic = 3, other medication = 1. ^#^ Time commitment = 18, not willing to undertake dietary intervention = 6, not willing to undertake trial procedures (blood tests, magnetic resonance imaging, body composition measurements) = 3, not willing to abstain from alcohol = 1. Taking anti-depressants = 2, hip replacement = 1.^^^ Time commitment = 8, not willing to undertake dietary intervention = 1, not willing to undertake trial procedure (magnetic resonance imaging) = 1, commenced a diet by themselves = 1. ^§^ History of abnormal liver function = 2, metal in body that was incompatible with magnetic resonance imaging = 5, chronic heart failure = 1, gastric banding = 6. Taking anti-depressants = 2, hip replacement = 1. BMI, Body Mass Index.

Participants were recruited from March 2013 to July 2016. The recruitment period resulted in a total of 2514 e-mail enquiries, out of which 401 (16.0%) proceeded to undertake a telephone screening (Figure 1). Of the 2113 enquirers who did not proceed to telephone screening, over half (56%) did not provide any further reasoning or correspondence. For the remaining 44%, reasons for not proceeding with their enquiry included self-reported ineligibility based on the 6 key trial criteria (see Methods, ‘Recruitment of potential participants’), no longer being interested in the trial due to the requirements of the trial, as well as medical conditions, medication or a history of gastric surgery, which were revealed after further email correspondence prior to arranging a telephone screening appointment with prospective participants. For the 401 individuals who proceeded to a telephone screening, 151 (6.0%) attended a face-to-face medical screening, and 101 (4.0%) were randomised (and enrolled) into the trial (Figure 1), fulfilling our original recruitment target of 100 women.

Of the 401 women who underwent telephone screening, almost two thirds did not proceed to face-to-face medical screening (*n* = [193 + 57 = 250]/401, 62.3%) (Figure 1), primarily due to exclusion for not meeting the eligibility criteria (*n* = 193/250, 77.2%). Of the women who did not meet the eligibility criteria, BMI was the most common reason for ineligibility (*n* = [32 + 16 = 48]/193, 24.9%) (Figure 1). At the face-to-face medical screening step, the main reason for not proceeding to enrolment into the trial was prospective participants’ refusal to participate (*n* = 32/[18 + 32 = 50], 64.0%) rather than exclusion for not meeting the eligibility criteria (*n* = 18/50, 36.0%). The main reason for refusal to participate, cited at the face-to-face medical screening (*n* = 11/32, 34.4%) as well as at the telephone screening (*n* = 28/57, 49.1%), was the inability to commit to the trial. Any other reasons are not known, as prospective participants ceased further correspondence after the telephone (*n* = 18) or face-to-face medical (*n* = 17) screening.

### 3.2. Recruitment Strategies

Different recruitment strategies were used concurrently throughout the recruitment period as summarised in Table 2. All but one strategy (print media, namely flyers) were utilised intermittently throughout the 2.5-year recruitment period and print media (flyers) were used throughout the 2.5 years.

### 3.3. Time or Monetary Cost per Randomised Participant, and Yield of Recruitment Strategies

To compare the overall success of different recruitment strategies, the time cost (labour) and monetary cost per randomised participant from each recruitment strategy was estimated, whereby lower values indicate greater cost-effectiveness and higher values indicate poorer cost-effectiveness (Table 2) [18,19]. The total number of prospective participants who made initial enquiries, were telephone screened, eligible, or recruited into the trial was also calculated for each recruitment strategy and is shown in Table 2, because yield is just as important as cost effectiveness. In the current trial, time cost per randomised participant is almost synonymous with the total time or monetary cost per randomised participant, because all recruitment strategies, except for one printed local newspaper advertisement in a circular entitled *MX* (AU$500) were free of monetary cost. Although printing of flyers and referral packs for healthcare professionals was provided by the University of Sydney, we estimated a cost of AU$905 for this print material (approximately 17,000 flyers × AU$0.05/flyer + 50 referral packs × AU$1.10/pack). In addition, the trial was supported by students and volunteers which significantly reduced the cost of hiring additional personnel. An estimated total of 828 h was spent on active recruitment, while approximately 1000 h (6–8 h/week) were spent managing e-mail and telephone enquiries and correspondences, including telephone screenings, each of which ranged from 5–60 min in duration. Assuming a casual pay rate of AU$40/h, the total cost of recruitment would have been AU$74,525 ([1828 h × AU$40/h] + AU$500 [for the local newspaper advertisement] + AU$905 [for printed materials]). Thus, over 98% of the costs for recruitment for this trial was the cost of time. While this equated to an average of 18 h (+AU$14 in advertisement and printing), or AU$738, invested for every participant randomised into the trial, the time cost per randomised participant varied between 0–122 h for different strategies (Table 2).

The recruitment strategy with the lowest time cost per randomised participant, and which was therefore the most cost-effective strategy, was word of mouth, which occurred passively and informally throughout the trial and consequently did not require any time or monetary investment for active recruitment and yielded 5 randomised participants.

The recruitment strategies with the equal-second-lowest time cost per randomised participant were free publicity on radio and TV, as well as printed advertorials (a sub-strategy of print media). Free publicity on radio and TV cost an average of 4 h per randomised participant and yielded a total of 15 randomised participants (radio *n* = 8; TV *n* = 7) (Table 2). It is noteworthy that radio publicity cost half as much as TV publicity (3 h per randomised participant versus 6 h for TV) and was used on twice as many instances as TV publicity (*n* = 6 versus *n* = 3, respectively) and received less than half as many responses as TV publicity (*n* = 17 versus *n* = 41, respectively). This difference is likely related to a greater proportion of respondents to radio than to TV publicity being eligible for the trial (47.1% versus 19.5%, respectively), as shown in Table 2. Like the average of radio and TV publicity, printed advertorials also cost an average of 4 h per randomised participant. This recruitment strategy yielded a total of 6 randomised participants.

Two recruitment strategies with intermediate time cost per randomised participant were internet-based strategies, and clinical trial databases and intranets (Table 2). Internet-based recruitment strategies yielded 24 randomised participants, which was the greatest of all recruitment strategies, although they cost an average of 23 h per randomised participant, which is almost six times greater than for free publicity on radio and TV or printed advertorials. Similarly, clinical trial databases and intranets cost an average of 22 h per randomised participant. This recruitment strategy only yielded 9 randomised participants.

The recruitment strategy with the overall greatest total time cost per randomised participant was flyers (a sub-strategy of print media), with an average cost of 35 h per randomised participant, albeit yielding 16 randomised participants, which is the second greatest yield of all strategies used. Approximately 17,000 flyers were distributed, equating to 1063 flyers per recruited participant. It is noteworthy that flyer distribution at community events, hospitals and the local tertiary education campus had intermediate time cost effectiveness, costing between 7–11 h per randomised participant. Also noteworthy is our observation that distributing flyers to local health service centres cost 61 h per randomised participant, while flyer distribution to residential mailboxes cost 122 h per randomised participant and only yielded 2 randomised participants.

Recruitment via referrals from healthcare professionals was not effective, incurring 24 h of investment but yielding 0 randomised participants (Table 2).

### 3.4. Level of Detail Provided in Recruitment Strategies and Impact on Time Cost per Randomised Participant

We hypothesised that recruitment strategies that provided greater detail about the trial (e.g., eligibility criteria and requirements) would have a lower time cost per randomised participant by enabling prospective participants to ‘self-screen’ prior to enquiring about the trial. Due to the nature of word of mouth information, the level of detail which was shared informally by individuals involved in the trial (researchers, students, participants, previous enquirers) and associates (family, friends, people who had learned about the trial through other recruitment strategies) could not be determined. For other recruitment strategies, the level of detail provided varied, mainly because media bodies decided on the final content. Free publicity on radio and TV only included detailed information about the trial in 2 of the 9 instances (22%), while printed advertorials included detailed information in 50% of instances, although both recruitment strategies were equal second in terms of the time cost per randomised participant. Internet-based strategies (which were intermediate in terms of the time cost per randomised participant) included detailed information about the trial in 6 of 37 instances (16%), and clinical trial databases and intranets (which had an intermediate time cost per randomised participant), flyers (which had the greatest time cost per randomised participant) and referrals from healthcare professional (which were not effective) provided detailed information about the trial in 100% of instances, as the trial researchers had complete control over the content in all copies. Taken together, these findings suggest that the level of detail provided in recruitment strategies did not influence the cost-effectiveness of a recruitment strategy.

## 4. Discussion

The current paper details the protocol for the TEMPO Diet Trial and describes and evaluates the effectiveness of recruitment strategies used to enrol participants into the trial. This trial found word of mouth to be the recruitment strategy with the lowest time cost per randomised participant, while free publicity on radio and TV, as well as printed advertorials, had the equal-second-lowest time cost per randomised participant. Despite being cost-effective, these recruitment strategies yielded only 26/101 participants. Internet-based and clinical trial databases and intranets had intermediate time cost per randomised participant, and yielded a further 33/101 participants. Another recruitment strategy that was intermediately cost-effective was flyer distribution at community events, hospitals and the local tertiary education campus, with a yield of 7/101 participants, while flyer distribution to local health service centres and residential mailboxes had the greatest time cost per randomised participant and yielded 6/101 participants. Recruitment via referrals from healthcare professionals was not effective. Taking into account both the cost-effectiveness of the different recruitment strategies and their yield, we conclude that clinical trials involving postmenopausal women with obesity could benefit from a combination of recruitment strategies, with an emphasis on word of mouth, free publicity via radio, TV and print media, and strategic placement of flyers, supplemented (if a greater yield of participants is needed) with internet-based strategies, databases and intranets.

Our finding that word of mouth was the most cost-effective recruitment strategy for women aged 45–65 years for the current randomised controlled trial of dietary obesity treatment extends previous findings in younger women with overweight or obesity, which showed that word of mouth was a more cost-effective recruitment strategy than radio, TV, printed advertorials and flyers, e-mails or mass snail mail [20]. The instances of reported word of mouth recruitments were sporadic in the current trial and did not increase over time, perhaps because strategic effort was not invested into this recruitment strategy. It is also possible that some participants who may have initially been referred to the trial by word of mouth were classified as having been recruited by other strategies, as they may have also learned about the trial via other recruitment avenues and reported these as their source of trial information. Future trials in this field could benefit from concerted efforts to enhance word of mouth recruitment alongside other strategies, as a highly cost-effective source of participant recruitment. While researchers in the current trial occasionally encouraged randomised participants and associates to share information about the trial, word of mouth could be further enhanced in future trials by actively providing trial details on distributable items. Such details could be included on items such as regular e-mailed newsletters to participants (including notification of up-coming public seminars by the research team on topics of relevance to weight loss), business cards, pens, and reusable folders and bags that participants and researchers could use as reference when discussing with others. However, it would be important to avoid overcapitalizing on trial-specific merchandise, as excessive spending in this domain would reduce the cost effectiveness of word of mouth recruitment.

Free publicity on radio and TV, as well as printed advertorials, were the equal-second most cost-effective strategies for recruiting older women with obesity into the current randomised controlled trial, possibly due to the nature of the announcements. Trial researchers leveraged long-term relationships with media contacts, including journalists and personnel in the university media office, to find opportunities for free publicity via avenues such as national radio, TV, magazines and newspapers that would otherwise be costly. Providing expert opinion on trial-related health news topics was a valuable form of reciprocity that further helped to develop collaborative relationships with media contacts. After announcements were made, contact was maintained with responsible media contacts (e.g., to give feedback on their media stories, to give thanks if an announcement generated a particularly strong number of enquiries about the trial, and to let them know that we were available for expert comment on any future media stories about weight management that they may be preparing), and this undoubtedly led to our ongoing opportunities for collaboration. Therefore, recruitment for future trials could benefit by establishing or maintaining long-term, strong, positive, mutually beneficial, collaborative relationships with media contacts, and soliciting opportunities for free publicity during recruitment drives.

Flyer distribution—overall—was the least cost-effective recruitment strategy in the current trial. While this is not true of flyers distributed to community events, hospitals and the local tertiary education campus, which had intermediate cost-effectiveness, it was true of distributing flyers to local health service centres and residential mailboxes. A previous study involving postmenopausal women in primary care interventions reported considerable success from residential mailbox flyers [9]. However, unlike our anonymous residential mailbox deliveries, that study used clinical cancer registries and senior community service organisation lists to address recruitment announcements to individuals who may have already held an interest in the topic of the trial [9]. Such registries may not be accessible to research groups due to privacy laws [9], and purchasing commercial lists of mailing contacts would be costly and would not guarantee an interested population. Although flyers were high in cost investment, not only in time for production and delivery, but also for printing materials, for future trials for this demographic, flyer distribution at community events, hospitals and local tertiary education campus could be specifically targeted, along with the other recruitment strategies listed above.

In the present trial, internet-based strategies and clinical trial databases and intranets also demonstrated intermediate cost-effectiveness. Internet-based strategies had a high influx of non-specific enquiries, which lead to a time cost per randomised participant that was almost 6 times greater than that of free publicity on radio and TV, or printed advertorials. Nonetheless, internet-based recruitment was a valuable strategy, as it contributed the largest number of randomised participants to the trial. For future trials, internet-based strategies could be used to supplement word of mouth recruitment and free publicity on radio and TV and printed advertorials, but could be better harnessed (and the cost-effectiveness improved) by implementing an automated response to direct initial enquirers to a web page from which they could learn more about the trial, including key eligibility criteria, and then—if still interested and if they consider that they meet these criteria—to register through an ethically-approved and secure online screening. Such automated systems would assist researchers to refine the sample of interested individuals they choose to contact based on pre-filled demographic and other details, thereby rendering this recruitment strategy more cost-effective. As mentioned above for trial-specific merchandise, it would be important not to overcapitalize on any such automated systems. Commercial options for automating and managing clinical trial recruitment are available to researchers, but these are often expensive. In contrast, many research organizations (e.g., universities, hospitals, and research institutes) have cost-effective bulk subscriptions to software that enables researchers to conduct surveys securely via the Internet, and these could be explored and cheaply deployed for clinical trial recruitment.

Contacting prospective participants through clinical trials databases and intranets was less cost-effective than expected. This may be explained by the lapse in time between prospective participants’ initial registration with the database, which may have been in response to announcement about the present or another clinical trial several months before contact by the research team or trial commencement. Evidence for this comes from one instance of free publicity on national TV, which had approximately 6 million viewers. While prospective participants were contacted within several days after their initial expression of interest, the trial commencement was not until five months or more afterwards, by which time ~58% of initial enquirers were either no longer interested or contactable.

Working through healthcare professionals to recruit participants via referrals yielded no contribution to the overall recruitment effort. Healthcare professionals may not have the time and resources to actively and conscientiously refer suitable patients to trials, and this method of recruitment may therefore be deprioritised for future trials unless they require clinical populations [5,9]. It should also be noted that the current trial invested less time and other resources into engaging and informing healthcare professionals about the trial, compared to free publicity on radio and TV, or print media and internet-based strategies or clinical trial databases and intranets. Thus, future trials, at least in those targeting participants with the demographic of the current trial, may benefit from minimising or completely avoiding recruitment via referrals from healthcare professionals.

The cost-effectiveness of the recruitment strategies implemented in the current trial could potentially be improved in future trials by including a trial telephone number, in addition to the e-mail address provided, to increase the number of initial enquiries. This is because people reading a printed article in a magazine or newspaper may find it easier to call a telephone number rather than to send an e-mail, by avoiding any delay that may be required to gain access to a device connected to the Internet. However, providing a trial telephone number would need to be supported by automated systems that do not lead to a greater time cost of recruitment, such as providing an automated message inviting prospective participants to leave a voice message with their mobile telephone number or e-mail address so that researchers can send participant information via short mobile messaging (SMS) or e-mail, including a link to conduct cost-effective surveys securely via the Internet as mentioned above.

This recruitment analysis has several strengths and limitations. To our knowledge, the current study is the first to evaluate the relative effectiveness of multiple recruitment strategies to engage and recruit postmenopausal women for a randomised controlled trial of dietary obesity treatments, as previous research has focused on younger adults [21,22]. However, the generalisability of the current results is limited to our target demographic, and results may differ for trials aiming to recruit people of other age or sex, or for different areas of clinical research. Time and monetary cost and cost-effectiveness were synonymous in the current study, because all strategies, except one paid newspaper advertisement, as well as printing costs which was provided by the University of Sydney, were free of monetary charge. This is because the current trial was well supported with students and volunteers, which significantly reduced the need to allocate limited government grant funding towards hiring additional personnel. In this sense, the current results are only applicable to other trials that also have access to the valuable contributions of students and volunteers, such as those conducted in universities, hospitals and research institutes. Hence, labour costs of recruitment for trials with limited access to students and volunteers were simulated using a casual pay rate for an average research assistant. A combination of cost effectiveness and yield of participants from different recruitment strategies need to be considered when researchers determine the overall suitability of recruitment methods for their specific research question, trial design and available resources. A further limitation of our recruitment time cost (or cost-effectiveness) calculations is that they do not predict conscientious and successful participants in terms of commitment and trial outcomes, as has previously been reported [22]. In addition, 40.1% of initial enquirers did not specify any details regarding their source of trial information, and others discontinued contact with the research team after the initial telephone screening invitation, rendering some self-reported recruitment data unobtainable—although it is likely that many of these enquiries were derived from Internet advertorials. Greater detail and rigour in documentation of recruitment data in future trials would reduce missing data and provide more refined insights into the recruitment process. Since the current recruitment analysis was conducted post-hoc, a randomised comparison of recruitment strategies was not conducted, and some avenues of recruitment were not fully assessed. While the trial incorporated some newer forms of popular media to recruit participants (Facebook, health blogs, Internet-based health articles), social media platforms and paid online advertisements were not explored in-depth, and may be subject to future investigation into the relative effectiveness of different Internet-based strategies. Moreover, commercial recruitment avenues were not explored at all in the current trial due to funding limitations. These may have resulted in faster recruitment. The current work is thus mostly relevant to research teams with limited access to research funds, but with strong access to students and volunteers, as is the case in many universities, hospitals and research institutes around the world.

## 5. Conclusions

The present findings highlight the inherent challenges of recruiting free-living older women with obesity into a randomised controlled trial of dietary obesity treatments, and illustrate a path forward for more cost-effective recruitment. In summary and conclusion, recruitment for future trials in this area will likely benefit by investing time into creative word of mouth recruitment strategies, building relationships with media contacts (including journalists and personnel in the university media office) to facilitate free publicity at the time when it is required for trial recruitment, only using flyers if they can be strategically placed in locations that the target demographic are known to utilise such as at community events, hospitals and local tertiary education campuses and—if recruitment yields with these three cost-effective strategies are not sufficient—internet-based strategies, all supported by automated online systems to reduce the time cost of screening to improve the cost-effectiveness of all recruitment strategies. These current results may provide important strategies and factors that could be considered in planning recruitment for future clinical weight loss trials.

## Figures and Tables

**Table 1:** Screening methods used to investigate inclusion and exclusion criteria for the TEMPO Diet Trial.

Inclusion Criteria	E-mail Screening	Telephone Screening	Face-To-Face Medical Screening
Female	X	X	X
45–65 years of age	X	X	X
Postmenopausal for ≥5 years (calculated from date of last menses)	X	X	X
Body Mass Index (BMI) 30–40 kg/m^2^	X	X	X
Weight stable (±2 kg) for ≥past 6 months		X	X
English-speaking		X	
Living in the Sydney metropolitan area (defined by the City of Sydney Statistical Division [11,12]) and able to attend all in-person appointments at the University of Sydney Camperdown campus	X	X	
Sedentary (defined as <3 h of structured physical activity per week)		X	X
Asked if they were capable of completing activities required for the trial (e.g., keeping a food, activity and sleep diary, wearing accelerometers for 7 days at a time, etc.)			X *
**Exclusion Criteria**			
Not ambulatory, or having restrictions to physical movement that would impede completion of trial activities		X	X
Osteoporosis			X
Extreme anaemia that could be exacerbated by the fast weight loss intervention (very low energy diet) to be used in the trial			X
Hyperthyroidism or hypothyroidism			X
Diabetes mellitus (defined by self-report during e-mail screening and by fasting blood glucose level ≥7.0 mmol/L and glycated haemoglobin (HbA_1c_) ≥ 6.5% at the face-to-face medical screening) [13]	X		X
Cardiovascular disease		X	
Gastrointestinal disease		X	
Previous gastric or other surgery that may affect appetite		X	
Any loose metal in the body (e.g., pacemaker or bullet) that is contraindicated for magnetic resonance imaging for safety reasons, or which may result in artefacts in medical imaging		X	X
Planning to undertake any major surgery in the next three years		X	
Tobacco use		X	
Alcohol or drug dependency		X	
Taking medication that affects heart rate, body composition or bone mass (e.g., beta-blockers, glucocorticoids)		X	X
Having taken anti-resorptive therapy within the last 3 years		X	X
Having taken medication that affects appetite, metabolism, or weight within the past 6 months		X	X
Any of the following contraindications for following a total meal replacement diet: lactose intolerance; following a strict vegan diet; or unwillingness to be randomised to one of the two diets		X	X
Donated whole blood within 3 months prior to trial commencement		X	
Liver or kidney impairments (which may render fast weight loss unsuitable)			X

* This was also verified prior to randomization and enrolment into the trial, which occurred 1 week prior to commencement of the dietary interventions (−1 weeks). X = applicable.

**Table 2:** Relative effectiveness of recruitment strategies used in the TEMPO Diet Trial.

Strategies	Description	Number of Instances Used	Total Response n (% of Total Response from all Strategies)	Screened n (% of Total Responses from Strategy)	Eligible n (% of Total Responses from Strategy)	Randomised n (% of Total Responses from Strategy)	Total Time Invested (Active Recruitment/Correspondence Time) (hours)	Time cost per Randomised Participant * (hours)
**All strategies**		**-**	**2514 (100.0)**	**401 (16.0)**	**151 (6.0)**	**101 (4.0)**	**1828 (828/1000)**	**18**
**Other** Word of mouth	Informally used throughout the trial through individuals directly involved with the trial (researchers, students, volunteers, ineligible participants and enrolled participants) and indirect associates (health practitioners, family, friends, acquaintances who may have learnt about the trial through the other recruitment strategies).	Continuous	29 (1.2)	17 (58.6)	11 (37.9)	5 (17.2)	10 (NA/10)	2
Not further specified	Individuals did not specify or could not recall their source of information in their initial enquiry.	-	1009 (40.1)	156 (15.5)	44 (4.4)	26 (2.6)	401 (NA/401)	15
**Free publicity on radio and TV (total)**	**TV and radio programs that invited trial researchers to provide commentary on health topics featured a free advertorial about the trial in exchange for accepting the interview. The suggested content of advertorials included brief description of the trial and trial e-mail address, which was announced verbally on air or as a web link on the channel website.**	**9**	**58 (2.3)**	**21 (36.2)**	**16 (27.6)**	**15 (25.9)**	**65 (42/23)**	**4**
Radio (local and national)	Trial recruitment information was mentioned on radio stations 2GB and ABC, during/at the end of segments involving guest researcher and was included on the radio station website.	6	17 (0.7)	8 (47.1)	8 (47.1)	8 (47.1)	25 (18/7)	3
TV (local and national)	Brief advertisement was included for stories about weight loss and health featuring trial researchers.	3	41 (1.6)	13 (31.7)	8 (19.5)	7 (17.1)	40 (24/16)	6
**Print media (total)**	**Printed advertorials in magazines and a local newspaper, and flyers.**	**-**	**78 (3.1)**	**61 (78.2)**	**40 (51.3)**	**22 (28.2)**	**588 (556/32)**	**27**
Printed advertorials (total)	Commercial advertisements included as part of magazine and newspaper articles featuring expert commentary from trial researchers. Advertisements were free of charge in exchange for providing expert commentary. Advertisement mentioned brief description of trial, key eligibility criteria and trial e-mail address, but was ultimately determined by the journalist or media liaison.	6	9 (0.4)	7 (77.8)	6 (66.7)	6 (66.7)	22 (18/4)	4
○ University magazine, newsletter	University of Sydney alumni magazine and newsletter.	2	4 (0.2)	4 (100.0)	3 (75.0)	3 (75.0)	8 (6/2)	3
○ Newspaper and magazine advertisements	Local and national newspaper and magazines, including one paid advertisement (AU$500) for a free local newspaper, entitled Mx, that was available to train commuters in Sydney metropolitan area.	4	5 (0.2)	3 (60.0)	3 (60.0)	3 (60.0)	14 (12/2)	5
Flyers (total)	A4 and brochure-sized flyers were printed using university facilities free of charge and distributed to locations in the Sydney metropolitan area by researchers, volunteers and students. The flyer included a brief description of the trial, key eligibility criteria and trial e-mail address. Over 17,000 flyers delivered in total, at AU$0.05 per flyer (total of AU$850).	-	69 (2.7)	27 (39.1)	17 (24.6)	16 (23.2)	566 (538/28)	35
○ Community events	Annual Sydney Craft & Quilt Fair held over 4 consecutive days (300 flyers delivered per day).	1	14 (0.6)	5 (35.7)	3 (21.4)	3 (21.4)	20 (14/6)	7
○ Hospitals	Hospitals in the Sydney metropolitan area, in waiting rooms, staff rooms and common areas.	Continuous	6 (0.2)	3 (50.0)	2 (33.3)	2 (33.3)	22 (20/2)	11
○ Local tertiary education campus	Libraries, common rooms and study spaces within the University of Sydney campus.	Continuous	5 (0.2)	3 (60.0)	2 (40.0)	2 (40.0)	22 (20/2)	11
○ Local health service centres	50–70 pharmacy and/or chemist sites (10 flyers delivered per site) and 100 medical practices (10 flyers delivered per site).	Continuous	8 (0.3)	4 (50.0)	4 (50.0)	4 (50.0)	243 (240/3)	61
○ Residential mailboxes ^	Houses and unit complexes (15,000 flyers delivered in total)	Continuous	10 (0.4)	4 (40.0)	2 (20.0)	2 (20.0)	244(240/4)	122
○ Not further specified	Individual did not specify or could not recall where they found flyer.	-	19 (0.8)	7 (36.8)	4 (21.1)	3 (15.8)	8 (NA/8)	3
○ Local community areas	6 libraries, 10–20 grocery stores/shopping centres, gyms and women’s centre (10 flyers delivered per site).	Continuous	7 (0.3)	1 (14.3)	0 (0.0)	0 (0.0)	7 (4/3)	NE
**Internet-based (total)**	**Free advertisements were featured in online health articles and social networking sites. Researchers requested a brief advertisement of the trial to be included in the footer of articles or as social media posts, including at least a brief description of the trial and e-mail address.**	-	**1082 (43.0)**	**128 (11.8)**	**40 (3.7)**	**24 (2.2)**	**541 (110/431)**	**23**
Health columns of news websites ^#^	Free advertisements were featured at the bottom of online health and nutrition-related articles of news and magazine sites which requested expert commentary from trial researchers.	37	1,033 (41.1)	127 (12.3)	40 (3.9)	24 (2.3)	521 (110/411)	22
Social media pages	Image of the trial flyer was posted on Facebook health pages related to women’s health and fitness.	1	32 (1.3)	0 (0.0)	0 (0.0)	0 (0.0)	13 (NA/13)	NE
Other	Individual read about trial through other online source which referred to an article or media release that advertised the trial.	Continuous	17 (0.7)	1 (5.9)	0 (0.0)	0 (0.0)	7 (NA/7)	NE
**Clinical trial databases and intranets**	**Advertisements and trial flyer were featured on staff intranets and portals of the University of Sydney, local tertiary hospitals (St Vincent’s Hospital and Royal Prince Alfred Hospital) and a local health network (Human Services Network). An ongoing advertisement was also listed on the University of Sydney clinical trials webpage which includes a database for research volunteers. Registrants in the database who met the trial criteria were invited by e-mail to arrange a telephone screening.**	Continuous	**248 (9.9)**	**40 (16.1)**	**17 (6.9)**	**9 (3.6)**	**199 (100/99)**	**22**
**Referrals from healthcare professionals**	**Personal letter invitations and trial information packs (10 flyers and a participant information sheet per pack) sent to healthcare professionals in medical clinics across Sydney metropolitan suburbs requesting referrals of suitable patients. Follow-up packs were sent to healthcare professionals or clinics that responded to initial invitations or referred individual(s). Participants enrolled into the trial provided details of referring healthcare professionals. ~50 professionals or clinics were sent packs, at AU$1.10 per pack (total of AU$55).**	Continuous	**10 (0.4)**	**5 (50.0)**	**0 (0.0)**	**0 (0.0)**	**24 (20/4)**	**NE**

All strategies were unpaid except one local newspaper advertisement which cost AU$500 and printing material which cost AU$905. NA = not applicable; NE = not effective, did not yield any randomised participants. * Time invested in active recruitment and correspondence (e-mails, telephone calls and telephone screenings) per randomised participant for each recruitment strategy, whereby smaller value indicates a more time effective strategy. Time costs are not additive per recruitment strategy category. ^#^ Health news column of websites including the Australian Broadcasting Commission, Huffington Post Australia, Body and Soul, News.com, Ninemsn, the Conversation, Yahoo7 and the Sydney Morning Herald. ^ Delivered to residential properties in the Northern Beaches, Inner West, Eastern and Inner Sydney metropolitan areas.
